# Pembrolizumab‐Associated Serositis With Recurrent Pleural Effusions Successfully Treated With Anakinra

**DOI:** 10.1002/ccr3.73112

**Published:** 2026-07-05

**Authors:** Emma McNally, Sean Garvey, Malcolm Herron, Niall Conlon, Sinead Cuffe, Richard Conway, Laura Gleeson

**Affiliations:** ^1^ Respiratory Department St James's Hospital Dublin Ireland; ^2^ Department of Clinical Medicine, School of Medicine Trinity College Dublin Dublin Ireland; ^3^ Clinical Immunology Department St James's Hospital Dublin Ireland; ^4^ Medical Oncology Department St James's Hospital Dublin Ireland; ^5^ Rheumatology Department St James's Hospital Dublin Ireland

**Keywords:** anakinra, immune checkpoint inhibitors, immune‐related adverse events, non‐small cell lung cancer, pleural effusions, serositis

## Abstract

Immune checkpoint inhibitor–associated serositis is rare and may present with recurrent effusions. Some cases are steroid dependent, contributing to significant toxicity for patients. Guidance on steroid‐sparing therapy is limited. Our case describes IL‐1 blockade with anakinra which was well tolerated and effective, supporting its use as a steroid‐sparing option.

## 1 Introduction

Immune checkpoint inhibitor (ICI)‐associated serositis is rare and remains poorly characterized. Management is largely extrapolated from more common immune‐related adverse events (irAEs), and evidence guiding steroid‐sparing therapy remains limited to case reports and small series. Here, we report a case of steroid‐dependent ICI‐related serositis with recurrent pleural effusions successfully managed with anti‐IL‐1 blockade.

## 2 Case History/Examination

A 70‐year‐old woman with T2aN3Mx lung adenocarcinoma (PD‐L1 40%) received carboplatin, pemetrexed, and pembrolizumab. Six‐month surveillance imaging revealed disease remission but new bilateral pleural and pericardial effusions, which over the following weeks progressed in parallel with breathlessness and generalized anasarca.

## 3 Differential Diagnosis, Investigations and Treatment

Laboratory testing showed B‐type Natriuretic Peptide (BNP) 450 pg/mL (< 900 pg/mL indicates congestive heart failure unlikely) albumin 30 g/L (reference range 30–50 g/L), elevated C‐reactive protein ranging from 55 to 107 mg/L (reference range < 5 mg/L) but white cell counts (WCC) within normal range (4–11 × 10^9^/L) and negative autoimmune serology. Echocardiography demonstrated preserved biventricular systolic function and whole‐body CT excluded recurrent malignancy. Bedside ultrasonography showed simple anechoic effusions, and thoracentesis confirmed sterile exudate with benign cytology. In the absence of infection, malignancy, or cardiac dysfunction, ICI‐related serositis was considered the most likely diagnosis.

**FIGURE 1:**
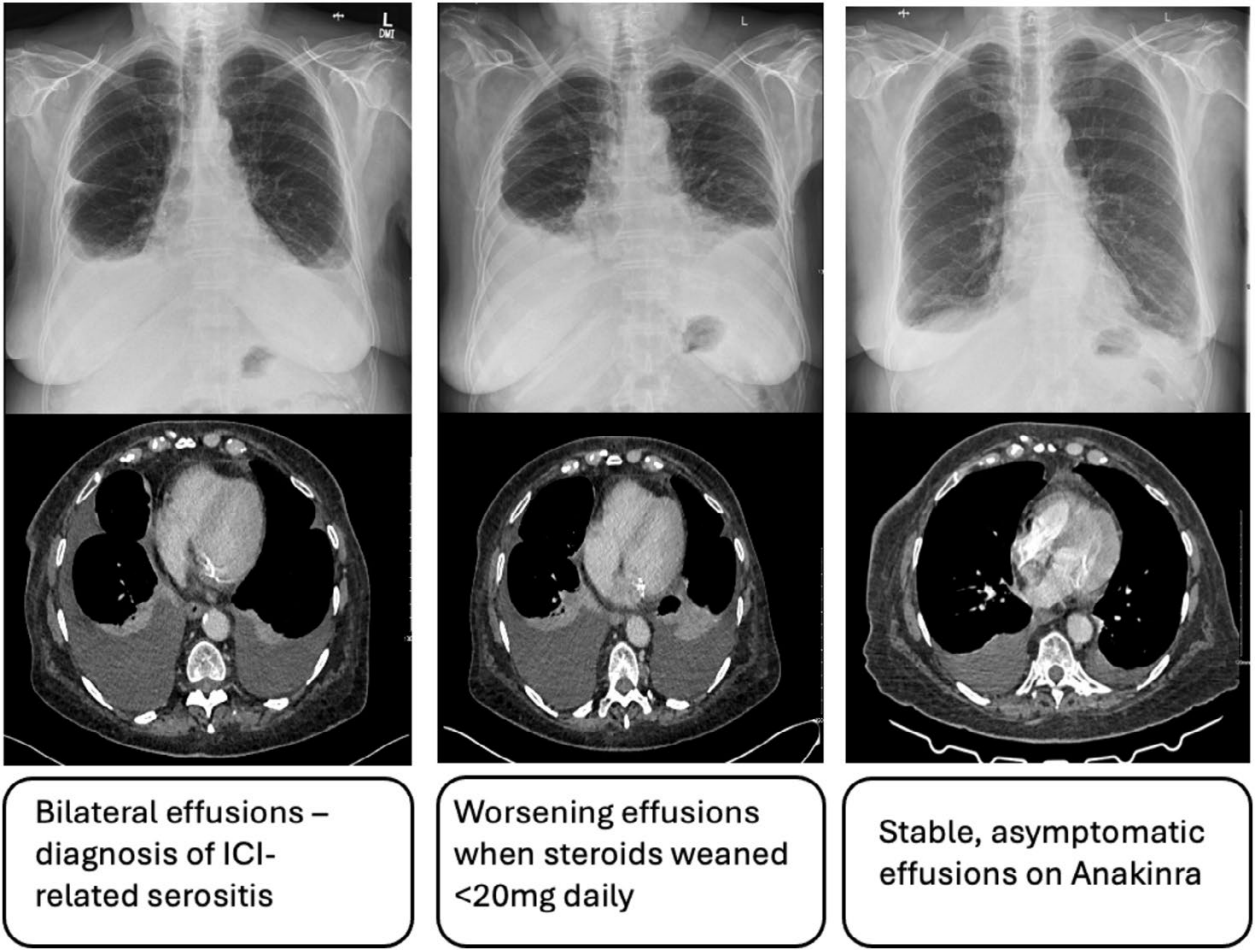
Serial chest radiographs and axial CT images show bilateral pleural effusions from immune checkpoint inhibitor–related serositis, worsening with corticosteroid taper and stabilizing after initiation of anakinra.

Pembrolizumab was discontinued and prednisolone 0.5 mg/kg initiated, resulting in symptomatic and radiological improvement. However, pleural effusions recurred consistently when prednisolone was tapered below 20 mg daily (Figure 1). Over the following 8 months, the patient underwent five thoracenteses, each yielding benign, exudative fluid.

Colchicine was trialed as a steroid‐sparing agent, initially at 0.5 mg three times daily, then reduced to twice daily and once daily in an attempt to improve tolerability. However, persistent gastrointestinal adverse effects led to discontinuation. Given ongoing steroid dependence causing progressive cushingoid features, immunosuppressive therapy was escalated.

Tocilizumab (anti‐interleukin [IL]‐6) was initiated based on reported efficacy in ICI‐related arthritis [1], allowing prednisolone tapering to 10 mg. However, symptomatic reaccumulation of effusions and widespread oedema occurred with further reduction, prompting discontinuation. Anakinra (anti‐IL‐1‐receptor) was commenced at 100 mg daily.

## 4 Conclusion and Results (Outcome and Follow‐Up)

Within 1 week, prednisolone was successfully reduced to 5 mg daily. At the time of writing, the patient remains clinically stable on anakinra and prednisolone 1 mg daily, with small asymptomatic pleural effusions and no ascites. Corticosteroid therapy is expected to be discontinued over the coming weeks (Figure 1).

## 5 Discussion

ICI‐mediated pleural effusion (IPE) remains ill‐defined and largely limited to case reports, typically presenting with bilateral effusions and may form part of a broader immune‐mediated serositis syndrome [2].

irAEs arise from removal of physiological inhibitory “checkpoints,” such as PD‐1/PD‐L1 or CTLA‐4, that normally restrain T‐cell activation. Their blockade enhances cytotoxic T‐cell activity and cytokine release against normal tissues, producing autoimmune‐type inflammation across multiple organs [3]. Reviews of irAE highlight contributions of NLRP3 inflammasome activation and macrophage recruitment in sustaining tissue‐specific inflammation [3, 4].

Corticosteroids are first‐line therapy for moderate‐to‐severe irAEs. Guidelines recommend prednisolone 0.5–1 mg/kg tapered over 4–6 weeks [5]. Our patient experienced reproducible recurrence of effusions below 20 mg prednisolone, indicating persistent serosal inflammation. Similar challenges have been described in a small series of ICI‐related polyserositis where two of four patients experienced partial response to steroids and required long‐term high‐dose steroids with repeated thoracocentesis [2].

Colchicine inhibits neutrophil chemotaxis and NLRP3 inflammasome activation [4] making it a biologically plausible adjunctive therapy. While evidence in ICI‐related serositis is limited, its established role in pericarditis supported its consideration. GI‐related adverse effects, however, rendered this option intolerable in this case [6].

A retrospective review of 92 patients with varying irAEs found that cytokine directed therapy such as IL‐6 blockade with tocilizumab or sarilumab was successful in 73% of cases, albeit this series did not include patients with polyserositis or pleural effusion [1]. In this case, response to tocilizumab was partial.

IL‐1 blockade using anakinra has shown efficacy in refractory polyserositis, with 32 of 37 patients reportedly steroid‐free in a recent multi‐center study [7] although this study did not include ICI‐associated serositis patients. The introduction of anakinra in our case resulted in rapid improvement in symptoms and allowed successful weaning of prednisolone below 5 mg with further dose reduction ongoing. Anakinra is an attractive option due to its proven long‐term safety and efficacy profile in other autoinflammatory conditions [7].

To our knowledge, this is the first report describing anakinra therapy for steroid‐dependent ICI‐associated serositis with recurrent pleural effusion, providing clinically relevant evidence to support steroid‐sparing strategies in refractory disease.

## Author Contributions

**Sinead Cuffe:** conceptualization, writing – review and editing, supervision. **Niall Conlon:** writing – review and editing, supervision, conceptualization. **Sean Garvey:** writing – review and editing, conceptualization. **Laura Gleeson:** conceptualization, writing – review and editing, supervision. **Emma McNally:** conceptualization, writing – original draft. **Richard Conway:** conceptualization, writing – review and editing, supervision. **Malcolm Herron:** conceptualization, writing – review and editing.

## Funding

The authors have nothing to report.

## Consent

Patient provided informed written consent to this report.

## Conflicts of Interest

The authors declare no conflicts of interest.

## Data Availability

Research data are not shared.

## References

[1] F. Fa'ak , M. Buni , A. Falohun , et al., “Selective Immune Suppression Using Interleukin‐6 Receptor Inhibitors for Management of Immune‐Related Adverse Events,” Journal for Immunotherapy of Cancer 11, no. 6 (2023): e006814. 37328287 10.1136/jitc-2023-006814 PMC10277540

[2] S. Zierold , L. S. Akcetin , E. Gresser , et al., “Checkpoint‐Inhibitor Induced Polyserositis With Edema,” Cancer Immunology, Immunotherapy 71, no. 12 (2022): 3087–3092. 35576074 10.1007/s00262-022-03211-7 PMC9588471

[3] B. Ibis , K. Aliazis , C. Cao , S. Yenyuwadee , and V. A. Boussiotis , “Immune‐Related Adverse Effects of Checkpoint Immunotherapy and Implications for the Treatment of Patients With Cancer and Autoimmune Diseases,” Frontiers in Immunology 14 (2023): 1197364. 37342323 10.3389/fimmu.2023.1197364 PMC10277501

[4] Y. Lu , J. Gao , Y. Hou , et al., “Targeting the NLRP3 Inflammasome Abrogates Cardiotoxicity of Immune Checkpoint Blockers,” Journal for Immunotherapy of Cancer 13, no. 1 (2025): e010127. 39773567 10.1136/jitc-2024-010127 PMC11749606

[5] V. R. Shannon , R. Anderson , A. Blidner , et al., “Multinational Association of Supportive Care in Cancer (MASCC) 2020 Clinical Practice Recommendations for the Management of Immune‐Related Adverse Events: Pulmonary Toxicity,” Supportive Care in Cancer 28, no. 12 (2020): 6145–6157. 32880733 10.1007/s00520-020-05708-2 PMC7471521

[6] J. Schulz‐Menger , V. Collini , J. Groschel , et al., “2025 ESC Guidelines for the Management of Myocarditis and Pericarditis,” Giornale Italiano di Cardiologia (Rome) 27, no. 1 Suppl. 1 (2026): e1–e93. 10.1714/4620.46292 41441823

[7] G. Lopalco , V. Venerito , A. Brucato , et al., “Anakinra Effectiveness in Refractory Polyserositis: An Italian Multicenter Study,” Joint, Bone, Spine 89, no. 2 (2022): 105299. 34656754 10.1016/j.jbspin.2021.105299

